# Random choices facilitate solutions to collective network coloring problems by artificial agents

**DOI:** 10.1016/j.isci.2021.102340

**Published:** 2021-03-24

**Authors:** Matthew I. Jones, Scott D. Pauls, Feng Fu

**Affiliations:** 1 Department of Mathematics, Dartmouth College, 27 N. Main Street, 6188 Kemeny Hall, Hanover, NH 03755, USA; 2 Department of Biomedical Data Science, Geisel School of Medicine at Dartmouth, Lebanon, NH 03756, USA

**Keywords:** Computer Science, Artificial Intelligence, Human-Computer Interaction

## Abstract

Global coordination is required to solve a wide variety of challenging collective action problems from network colorings to the tragedy of the commons. Recent empirical study shows that the presence of a few noisy autonomous agents can greatly improve collective performance of humans in solving networked color coordination games. To provide analytical insights into the role of behavioral randomness, here we study myopic artificial agents attempting to solve similar network coloring problems using decision update rules that are only based on local information but allow random choices at various stages of their heuristic reasonings. We show that the resulting efficacy of resolving color conflicts is dependent on the implementation of random behavior of agents and specific population characteristics. Our work demonstrates that distributed greedy optimization algorithms exploiting local information should be deployed in combination with occasional exploration via random choices in order to overcome local minima and achieve global coordination.

## Highlights

• Local information makes solving distributed network coloring problems difficult
• Greedy agents can become gridlocked, making it difficult to find a global solution
• Agents making random choices can facilitate the finding of a global coloring
• Randomness can be finely tuned to a specific underlying population structure

## Graphical abstract

**Figure:**
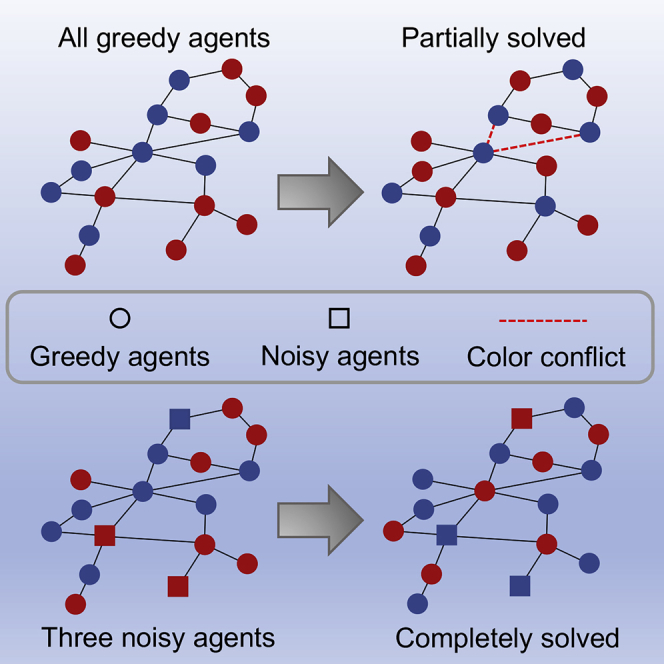

## Introduction

Many classical games like the Prisoner's Dilemma focus on two players attempting to get the better of each other. Both players would like to defect while their opponent cooperates, thus reaping rewards and avoiding punishments. A great body of work is focused on how to foster cooperation in such non-zero sum games (Nowak, 2006b; Doebeli and Hauert 2005). But there is another well-studied class of games in which all players receive the most benefit when they work together, called coordination games (Skyrms 2004). The optimal behavior for all players can be easily determined and agreed upon if all players can meet and strategize beforehand. In such games, the difficulty comes not from attempting to scam one's opponent but figuring out what one's partner will play before choosing one's own strategy (Huyck et al., 1990; Nowak 2006a). However, there can still be a “defecting” component, in which one's opponent can unilaterally choose a strategy with lower maximum payoff but also less risk (Fang et al., 2002).

Frequently, we consider playing games in which the population is given some spatial structure other than being well mixed (Durrett and Levin 1994; Szabó et al., 2005). Population structure is typically modeled as a graph or network, where each node is an individual, and individuals play games if they are connected by an edge (Ohtsuki et al., 2006; Santos and Pacheco 2005; Fu et al., 2008; Perc and Szolnoki 2010; Rand et al., 2011; Shirado et al., 2013; Gómez-Gardenes et al., 2007; Shirado and Christakis 2020). On such a network, many coordination games can be rephrased as network coloring problems (Judd et al., 2010). A coloring is a collection of labels or colors, one for each node, such that any two nodes connected by an edge have different colors. Network colorings make appearances in all sorts of fields, including sudoku puzzles, register allocation in computer science (Chaitin 1982), and clustering problems (Hansen and Delattre 1978). Deciding on a time table for various classes with shared classrooms (Werra 1985) and assignment of radio frequencies (Zoeliner and Beall 1977) are just two examples or coordination games that manifest naturally as network coloring problems. Generally, we let the nodes be individuals (which we refer to as artificial agents in this work), and the color choice represents the strategy of that individual. When the nodes of a network are properly colored, all the individuals are playing an optimal strategy. In this sense, the network coloring problem, if assigned with a proper payoff structure for the coloring outcome, can be considered broadly as a coordination game (Kun et al., 2013; Apt et al., 2014).

In general, the network coloring problem is non-deterministic polynomial-time-hard (Garey and Johnson 1999). It is found that many difficult mathematical problems cannot be solved by a simple, direct approach, but it can help to apply a small degree of randomness to any algorithms searching the solution space. This approach has been used to all sorts of problems, including the Traveling Salesman Problem (Bonomi and Lutton 1984) and the graph coloring problem (Johnson et al., 1991) with which we are concerned in this paper. It is noteworthy that, more broadly, effects of noises on phase transitions and collective outcomes have been studied in diverse contexts, including consensus in opinion formation and evolution (Pineda et al., 2009; Su et al., 2017), ordering in Kawasaki dynamics (Kawakatsu et al., 1993), cooperation in evolutionary games (Perc 2006; Szolnoki et al., 2009), and convergence in combinatorial optimization problems (Cai et al., 2020), just to name a few.

Attempts to solve the network coloring problem typically use information about the entire network to make decisions about the colors of nodes. This makes sense as having all the information simultaneously leads to better informed decisions. For example, Johnson et al., 1991 use a notion of temperature to gradually reduce stochastic behavior as the system “cools” into the global solution. This requires some central information unit that instructs each node on color choice. However, if we are using the network as a model of a population in which edges represent interactions, such a central “brain” may not exist. Instead, individuals may be forced to make decisions based on nothing except the color of their neighbors at any given moment. Thus, solving the distributed network coloring problem, in which each node decides its color with only the local information about its neighbors, is more difficult, as we lose the ability to make decisions based on the state of the entire network.

In recent years, there has been a growing interest in studying distributed coloring problems. One line of work involves deterministic algorithms that require more colors than necessary for the network (Finocchi et al., 2004; Chaudhuri et al., 2008). The additional available colors make the problem much more tractable. There has also been work involving experiments with human subjects who have been given control of the color of a single node and are asked to choose colors to eliminate conflicts with their neighbors. Kearns 2006 observed that individuals would frequently choose colors that temporarily increased the total number of color conflict but ultimately lead to a global coloring. Shirado and Christakis 2017 found that by adding a small number of bots (namely, artificial agents as opposed to humans) to the system who periodically made random changes “decreased both the number of conflicts and the duration of unresolvable conflicts” when finding network colorings. However, they also found that the bots could be detrimental if not properly tuned with the appropriate levels of randomness (Shirado and Christakis 2017). Along this line, a recent related modeling work has incorporated reinforcement learning algorithms (q-bots) into agent-based simulations of the distributed coloring problem (Qi et al., 2019). Despite these developments, there still is a lack of analytical insights into the optimal level of random behavior needed when solving network coloring problems.

To provide further analytical insights into the role of behavioral randomness (Kearns 2006; Shirado and Christakis 2017), here we study myopic artificial agents attempting to solve similar network coloring problems using decision update rules that are only based on local information but allow random choices at various stages of their heuristic reasonings. Without loss of generality, we assume agents are situated on the simple case of networks that can be colored with only two colors, often called bipartite networks (Guillaume and Latapy 2006). This specific network structure simplifies the number of possible colorings (exactly two for a connected network) and offers analytical insights that would be formidable to obtain otherwise. The results reported below come from an entire population of artificial agents (in the fashion of simulated bots as in the study by Shirado and Christakis 2017), some of whom are behaving deterministically and some stochastically. Our work sheds some light on the appropriate levels of randomness to optimize solving the distributed coloring problem.

## Results

### Random network construction

As we will see, different network topologies will be easier or harder to color. Even with global information, finding network colorings becomes exponentially more difficult as the number of nodes increases (Garey and Johnson 1999). On the other hand, as average degree increases, individuals will have more neighbors and therefore be able to make more informed decisions when choosing a color. Throughout this paper, we simulate artificial agents that attempt to find 2 colorings of random bipartite networks. The exact structure of these networks will vary, as will the decision update rules agents use to solve the network colorings.

We construct a random network with *n* nodes and average degree *k* by first assigning each node to group A or group B with probability ½. Then, we add an edge between any two nodes in different groups with probability 2*k*/*n*. Thus, the resulting network is guaranteed to have a 2-coloring solution by assigning every node in group A one color and every node in group B the other color. However, there may be different numbers of nodes for each color, as the sizes of groups A and B are binomially distributed in our bipartite network model.

### Decision update rules for agents

In this paper, we consider multiple decision update rules to account for a variety of artificial agents' behavior, each with their own strengths and weaknesses. In the following, an “acceptable” local coloring at a node is the choice of color such that none of the node's neighbors have that color (no color conflicts with neighbors).

We first consider a basic “greedy” update rule of agents:

#### I: Basic greedy update rule

Step 1: Check if the current color is already an acceptable local coloring. If yes, keep the current color for this update step. If not, advance to step 2.

Step 2: Check if the other color would make an acceptable local coloring. If yes, change to that color. If not, advance to step 3.

Step 3: Choose whichever color will minimize the number of color conflicts. If both colors will create the same number of color conflicts with neighbors, randomly choose one color.

As the goal of each agent is to reduce and ultimately eliminate color conflicts with their neighbors, the greedy update rule can be seen as the “rational” strategy for an agent playing every single round of the coloring game and is therefore implemented as the “default” strategy in our population. We incorporate random behavior in various decision stages in the following modified update rules based on the basic greedy update rule above. Notably, these simple yet natural update rules based on intuitive heuristics, combined with the bipartite network structure which simplifies the possible colorings, enable analytical insights that are unobtainable in the more complicated systems put forward in other work (Qi et al., 2019).

#### II: Randomness-first update rule

Step 1: With probability *p*, choose a color uniformly at random. With probability 1−*p*, advance to step 2.

Step 2: Check if the current color is already an acceptable local coloring. If yes, keep the current color for this update step. If not, advance to step 3.

Step 3: Check if the other color would make an acceptable local coloring. If yes, change to that color. If not, advance to step 4.

Step 4: Choose whichever color will minimize the number of color conflicts. If both colors will create the same number of color conflicts with neighbors, randomly choose one color.

#### III: Memory-0 update rule

Step 1: Check if the current color is already an acceptable local coloring. If yes, keep the current color for this update step. If not, advance to step 2.

Step 2: Check if the other color would make an acceptable local coloring. If yes, change to that color. If not, advance to step 3.

Step 3: With probability *p*, choose a color uniformly at random. With probability 1−*p*, advance to step 4.

Step 4: Choose whichever color will minimize the number of color conflicts. If both colors will create the same number of color conflicts with neighbors, randomly choose one color.

#### IV: Memory-*N* update rule

Step 1: Check if the current color is already an acceptable local coloring. If yes, keep the current color for this update step. If not, advance to step 2.

Step 2: Check if the other color would make an acceptable local coloring. If yes, change to that color. If not, advance to step 3.

Step 3: If no neighbors have changed colors in prior *N* cycles, with probability *p*, choose a color uniformly at random, and with probability 1−*p*, advance to step 4.

If any neighbors have changed colors in prior *N* cycles, advance to step 4.

Step 4: Choose whichever color will minimize the number of color conflicts. If both colors will create the same number of color conflicts with neighbors, randomly choose one color.

### Initialization of agent behavior

Each artificial agent, located at a node in the network, behaves according to one of the aforementioned update rules. Specifically, we consider scenarios where the population may be using two different update rules. A certain fraction *ρ*_*r*_ of randomly selected agents adopt one of the randomness-first, memory-0, or memory-*N* update rules where the propensity of random behavior is *p* (as defined in the update rules), and the rest of agents use the basic greedy update rule.

The color choice of agents is updated in a random sequential manner (Szabó and Fath 2007). Agents update one at a time, and the order in which agents update is random. Each agent begins with a randomly chosen color.

### Difficulty metrics

We use three different metrics to quantify how successful a given decision update rule is in solving coloring problems by artificial agents: the number of unsolved networks, the number of update cycles, and the number of player updates.

The number of unsolved networks metric is simply the probability that a given network will reach a coloring given certain initial conditions including update order, update rules for each agent, and initial coloring.

The number of update cycles measures the number of times each agent goes through the update process, and the number of updated agents measures the total number of color changes. Roughly, the number of update cycles measures how long it will take the system to reach a coloring in real time, and the number of updated agents measures how involved the process is for all agents involved. Because some combinations of networks and initial conditions may never end up with a complete coloring solution, these metrics have the possibility to be infinite in these cases. Therefore, the average of difficulty metrics across model parameter combinations may be heavily skewed by some of the unsolved network coloring cases. Nevertheless, these difficulty metrics provide a practical means to compare the efficacy of resolving color conflicts across simulated scenarios and can help reveal interesting results to some extent.

### Bowties and gridlock

**Figure 1:**
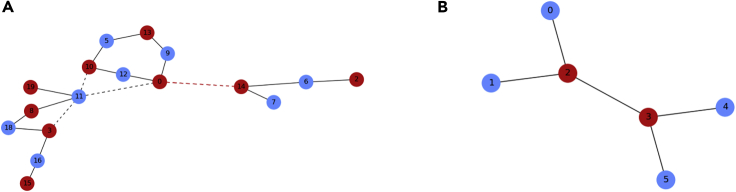
Overcoming local minima is often needed to solve collective action problems (A) shows a small network that did not find a valid coloring using only greedy behavior. The four dashed edges represent “bowties,” subgraphs where the greedy update rule can become gridlocked. The red edge shows a color conflict that cannot be resolved by greedy behavior. In (B), we see how the interior nodes of a bowtie are both forced to keep the same color by the exterior nodes, creating gridlock.

To see how local minima arise, we show a small network in which each agent occupying a network node uses the greedy update rule in Figure 1A. The dashed edges are “bowties,” small subgraphs consisting of a central edge whose end nodes both have at least three edges. Motif structures like this can lead to gridlock and the failure of the greedy update rule, as demonstrated in Figure 1B. If the central agents are playing the same color, they can become locked in by their other neighbors, and as a consequence, the greedy update rule becomes trapped at this local minimum, unable to explore the entire space and find a global minimum of color conflicts. Without random behavior, the network will never reach a global coloring once this happens. The smallest possible network structure that can become gridlocked is the six-node bowtie, as shown in Figure 1B.

This simple case demonstrated in Figure 1B can yield an interesting insight. Consider the case where there is no random behavior and each agent is playing the greedy update rule. There are 6!⋅2^6^ random initial conditions for the update order and initial colors. Using exhaustive search to work out each case, we find that the simple bowtie results in gridlock with probability 29/120. In each case, either gridlock or a global coloring is always reached after two update cycles.

Of course, brute-force computation quickly becomes untenable for large network sizes, but we can still develop helpful intuition from this simple example (Figure 1B). With the randomness-first update rule, if at least one agent has random behavior (occurs with probability 1−(1−*ρ*_*r*_)^6^), the network will eventually find a global coloring. However, in the memory-*N* update rule, the peripheral nodes already have a locally acceptable color and will not change even if they have the potential for random behavior. One of the middle two nodes must have random behavior to find a coloring, which happens with probability 1−(1−*ρ*_*r*_)^2^, a much less likely event than in the randomness-first update rule. Thus, the gridlock probabilities for the randomness-first and memory-*N* update rules respectively are approximately given as follows:

Equation (1) $$P_{\text{rand}-\text{first}}\left(\text{Gridlock}\right)=\frac{29}{120}{\left(1-\rho_{r}\right)}^{6}$$

Equation (2) $$P_{\text{memory}-N}\left(\text{Gridlock}\right)=\frac{29}{120}{\left(1-\rho_{r}\right)}^{2}$$

**Figure 2:**
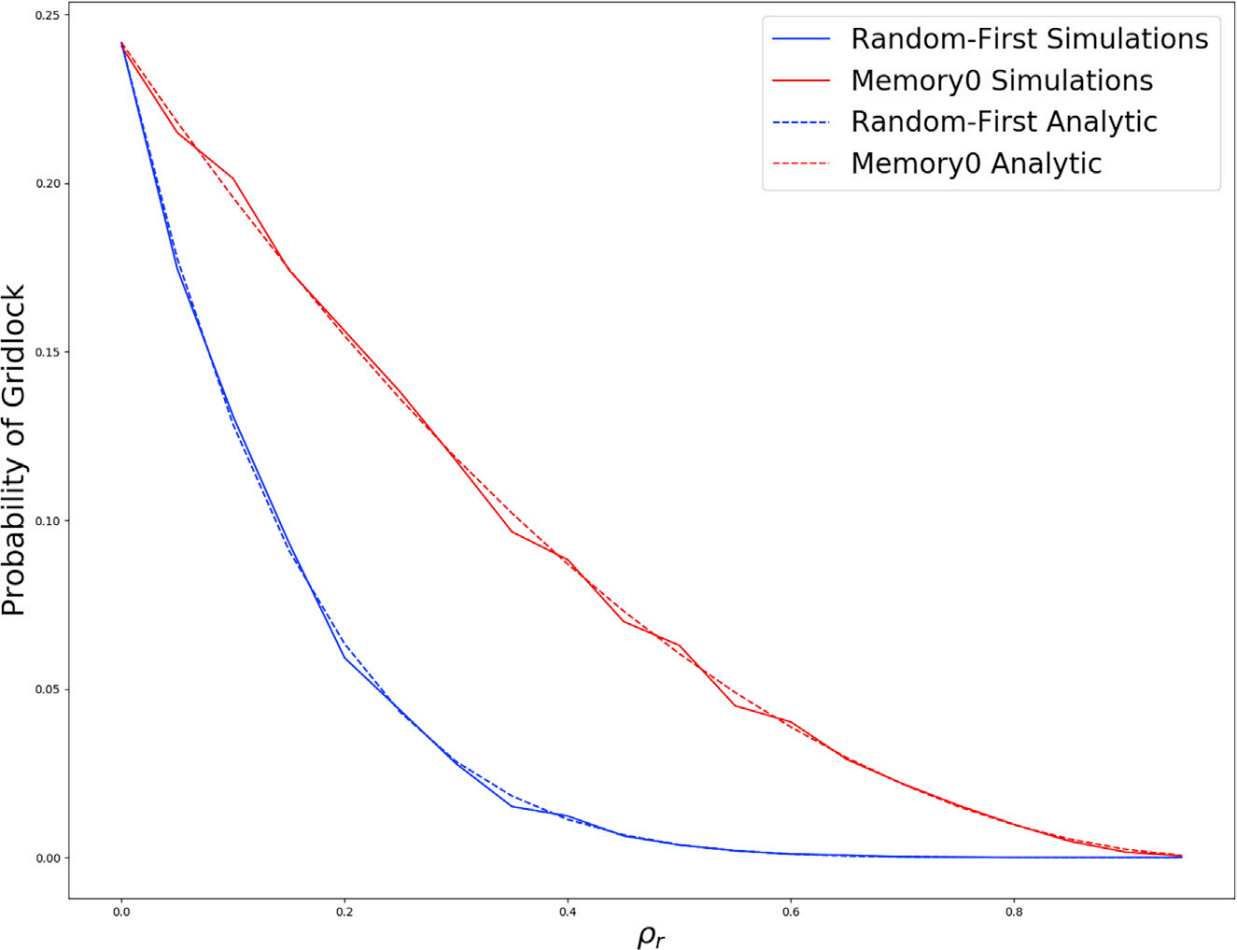
The probability of gridlock in the six-node bowtie for varying the fraction of agents with random behavior, *ρ*_*r*_. We see that the simulations (using *p* = 0.5) match well with the analytic results in Equations Equation (1), Equation (2). Here, we compare the randomness-first rule with the memory-0 rule. Simulation results are averaged over 1,000 independent runs.

We see excellent agreement between these equations and simulations in Figure 2. We note that these probabilities are less accurate when *p* is large because individuals could behave randomly before the system reaches gridlock, disrupting the earlier computation for 29/120 which assumed no random behavior takes place in the first two update cycles.

Similarly, we see that the memory-*N* update rules require larger *ρ*_*r*_ than the randomness-first rule to reach the same efficacy of resolving color conflicts. When using the former update rule, only agents with a color conflict are allowed to make random choices, unlike the latter randomness-first update rule. Because random behavior is limited to individuals with a color conflict, large *ρ*_*r*_ values are less likely to result in too much randomness when most agents are already in a local coloring without conflicts and hence will not behave randomly in any given time step. We shall see this difference between randomness-first and memory-*N* update rules manifest itself in simulations on larger networks in the following section.

### Monte Carlo agent-based simulations

**Figure 3:**
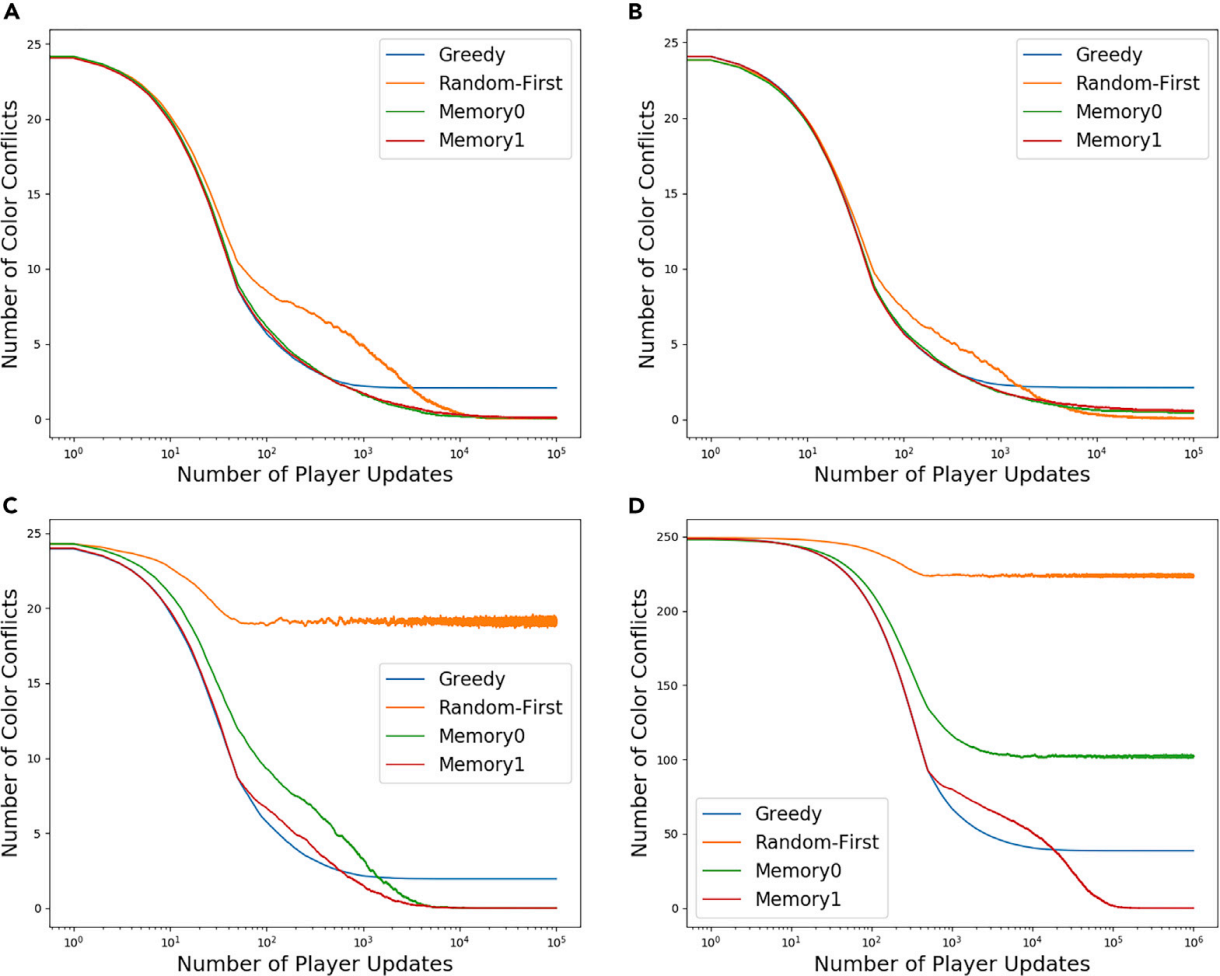
Plots of total conflicts vs time. Each curve is the average of 1,000 simulations, and each run consists of 2,000 update cycles Observe that the x axis is log-scale to show the short- and long-term behavior of each update rule. All networks have average degree 2, and the other network properties are as follows: (A) *n* = 50,*p* = 0.1,*ρ*_*r*_ = 0.9, (B) *n* = 50,*p* = 0.1,*ρ*_*r*_ = 0.5, (C) *n* = 50,*p* = 0.6,*ρ*_*r*_ = 1, (D) *n* = 500,*p* = 0.6,*ρ*_*r*_ = 1

Having defined the model parameters for the problem, we now can ask a basic question: What is the optimal amount of randomness to have in the system so as to reach a coloring solution? It turns out that the answer varies, depending on the specific update rule used, the size of the network, and the average degree of the underlying network. Typically, we will consider large and small networks with 50 and 500 nodes and vary with average network degree values of 2 and 20, respectively. Figure 3 shows how noisy agents using different update rules succeed at reducing the total number of conflicts in different situations. Notice that no update rule alone can beat the greedy update rule in the short term, but eventually, the randomness-based update rules begin under-performing the greedy rule only to eventually surpass it and completely eliminate color conflicts.

There are two sources of difficulty for coloring networks using any randomness-based update rule. If there is not enough randomness, the decision update rule is unable to break away from the local minimum found by agents using the greedy update rule. If there is too much randomness, the probability that at least one agent will be picking the wrong color every turn is so high that the network will not find a coloring in a reasonable number of time steps. Methods like simulated annealing avoid this problem by cooling the system and decreasing the amount of randomness over time (Johnson et al., 1991). However, in a distributed system (where each agent is using only local information to choose color) with no global information like temperature, we are limited to very simple local update rules that simply cannot evolve over time.

### Randomness-first rule

**Figure 4:**
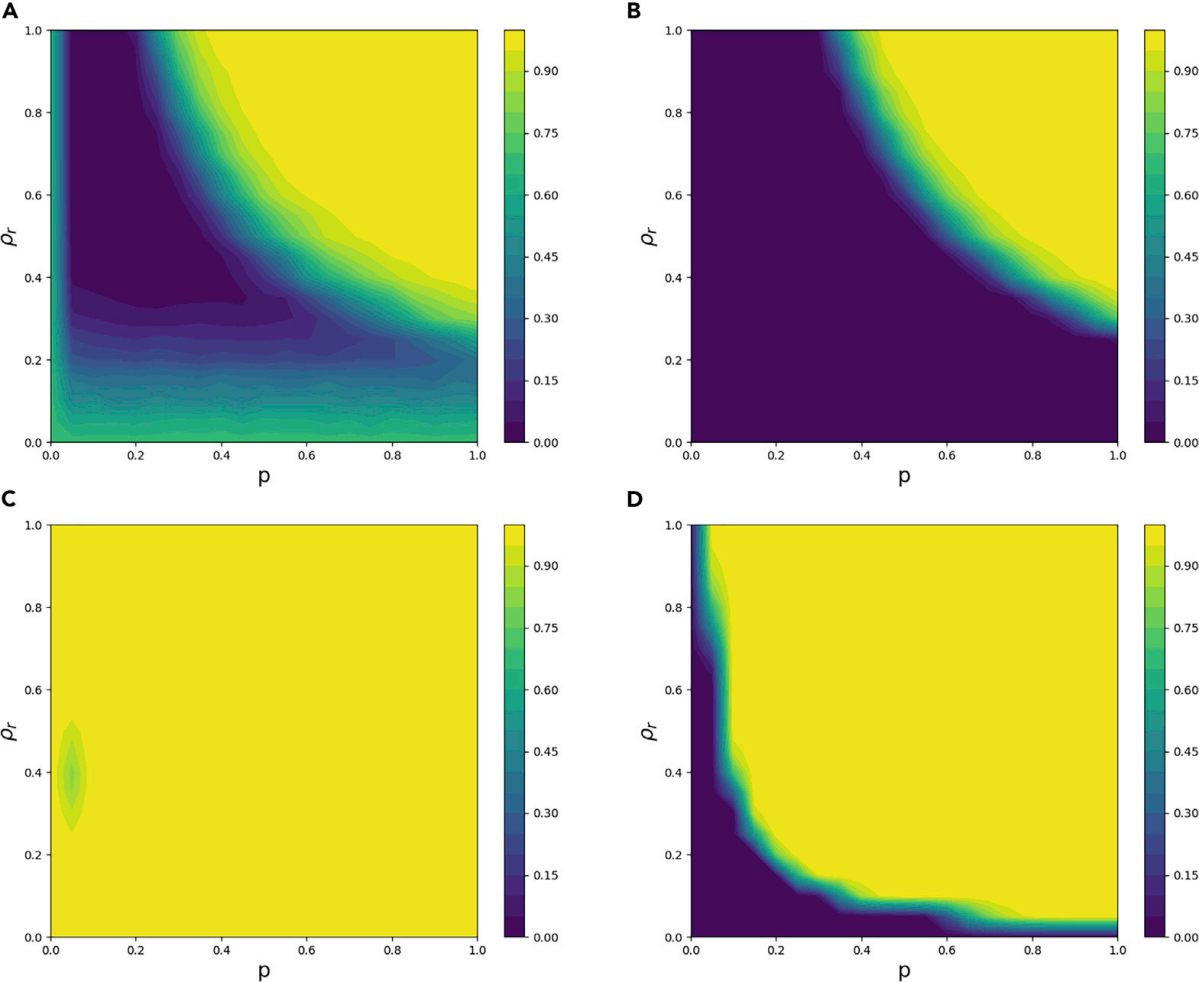
For the randomness-first update rule, simulation results of the probability of not solving the network in 10,000 time steps using four different types of networks as a function of the level of randomness *p* and the fraction of agents with random behavior *ρ*_*r*_ The bipartite network parameters including the size *n* and the average degree *k* used for the underlying networks are as follows: (A) *n* = 50,*k* = 2, (B) *n* = 50,*k* = 20, (C) *n* = 500,*k* = 2, (D) *n* = 500,*k* = 20.

For the randomness-first update rule, we ran simulations for 20 combination values of *ρ*_*r*_ and *p* between 0 and 1. Networks that found a coloring within 10,000 update cycles by agents were considered solved, and those that did not find a coloring within 10,000 cycles were considered unsolved. In Figure 4, we show the results of these simulations.

We see the difficulty of too much and too little randomness in Figure 4. In all four regions of the network parameter space (small/large size, low/high edge density), the probability of solving the network goes to zero because agents are always making random decisions, even when the rest of the network has found a local coloring. When average degree is two, we also see unsolved networks when there is very little randomness. Namely, there are too few random agents to break out of the local minimum.

These results demonstrate how the randomness-first update rule's success varies depending on the properties of the network (Figure 4). When average degree is high, randomness is actually a hindrance; the fewer random actions there are, the better. However, when average degree is low, a large fraction of the population using the randomness-first update rule with a low *p* is best. Unfortunately, for large networks with small average degree, there seems to be no good *p* and *ρ*_*r*_ when using the randomness-first rule.

Notice that in general, as network size goes up and/or average degree goes down, there are more unsolved networks. This makes intuitive sense, as the presence of additional nodes means more colors that need to be correct, and smaller average degree means the nodes have less information and make poorer decisions.

### Memory-*N* rules

**Figure 5:**
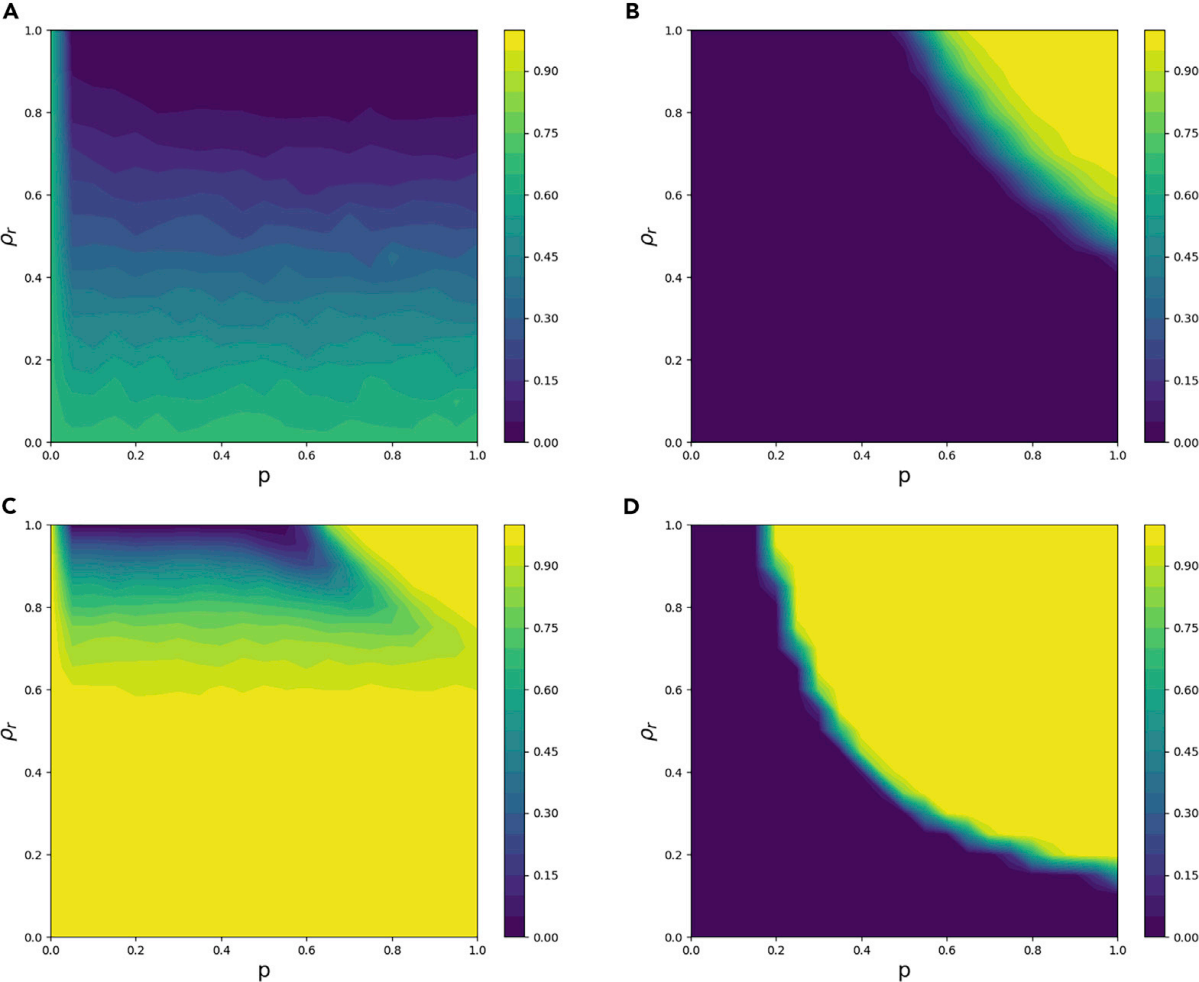
For the memory-0 update rule, simulation results of the probability of not solving the network in 10,000 time steps using four different types of networks as a function of the level of randomness *p* and the fraction of agents with random behavior *ρ*_*r*_ The bipartite network parameters including the size *n* and the average degree *k* used for the underlying networks are as follows: (A) *n* = 50,*k* = 2, (B) *n* = 50,*k* = 20, (C) *n* = 500,*k* = 2, (D) *n* = 500,*k* = 20.

We first study the memory-0 update rule that differs from the randomness-first rule in that agents only take random actions if they are in conflict with at least one of their neighbors. Thus, there are fewer needless random actions, and we would expect this decision update rule to perform better where excess randomness is an issue. This is partially confirmed by simulations in Figure 5.

**Figure 6:**
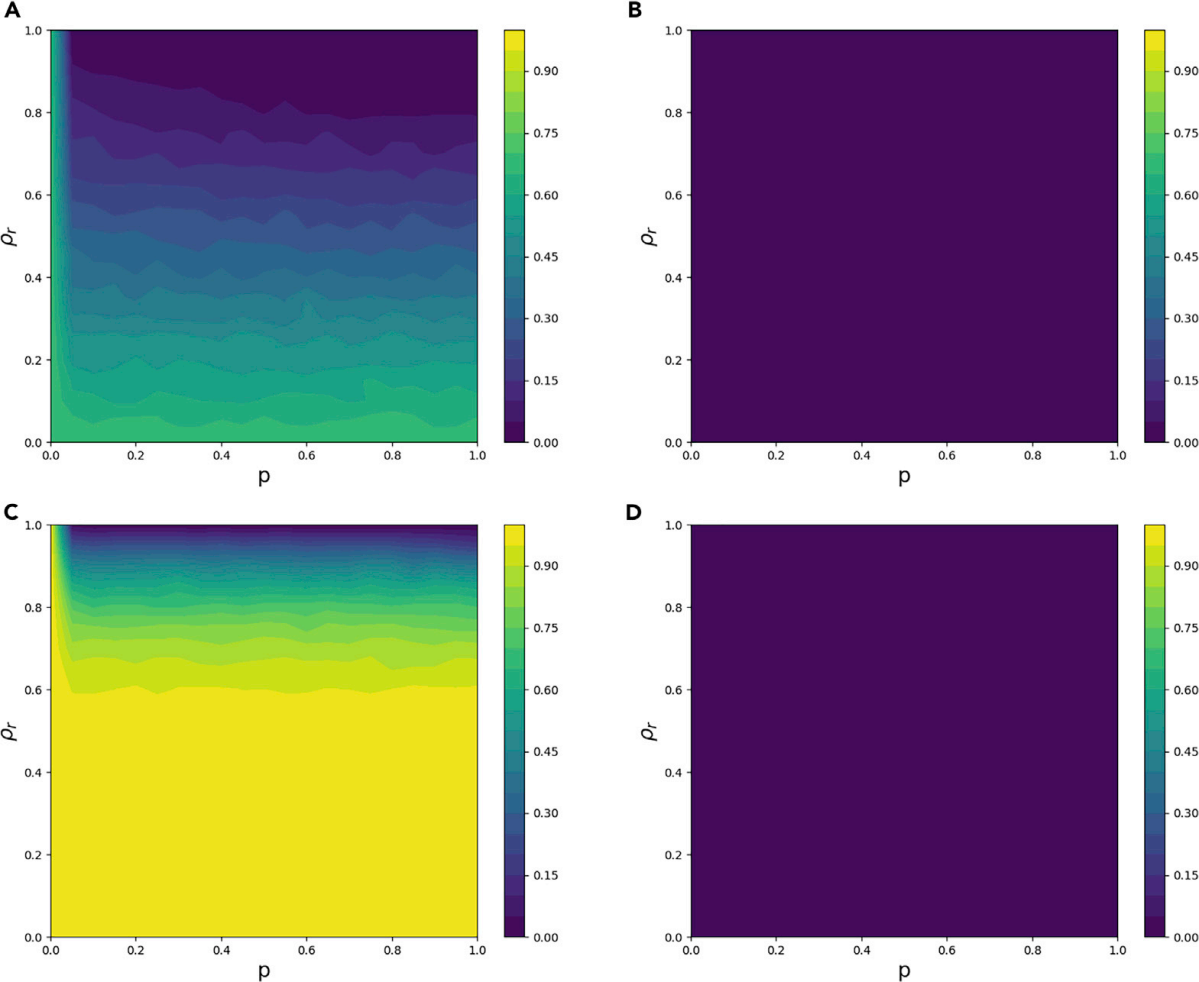
For the memory-1 update rule, simulation results of the probability of not solving the network in 10,000 time steps using four different types of networks as a function of the level of randomness *p* and the fraction of agents with random behavior *ρ*_*r*_ The bipartite network parameters including the size *n* and the average degree *k* used for the underlying networks are as follows: (A) *n* = 50,*k* = 2, (B) *n* = 50,*k* = 20, (C) *n* = 500,*k* = 2, (D) *n* = 500,*k* = 20.

Generally, we see an improvement of performance over the randomness-first update rule. The memory-0 rule does very well when *ρ*_*r*_ is close to one, even for large networks with low average degree. However, it still struggles with excess randomness, particularly when network size and average degree are large. A higher average degree means that a single random color choice creates more color conflicts and therefore makes it more difficult for the system to settle into a global coloring. However, if we assume agents with a longer memory (i.e., *N* ≥ 1), this issue vanishes, as demonstrated in Figure 6.

This compelling evidence suggests that the memory-1 update rule is most effective at resolving color conflicts as compared with the basic greedy update rule and the memory-0 update rule (cf. Figures 4, 6, and 5). If *ρ*_*r*_ is close to one, networks are almost always able to find a global coloring, regardless of network size or average degree. However, if for some reason only a rather small fraction of the agents are allowed to use randomness-based update rules, the randomness-first update rule will have more success, as seen in the simple bowtie example in Figure 1B.

The memory-1 update rule is extremely effective in networks with high connectivity. Similar effects of connectivity on graph colorablity, albeit using the Brélaz's heuristic algorithm (Brélaz 1979), have been observed in coloring small-world networks (Svenson 2001). When average degree is *k* = 20, every individual's local information encompasses the color of a large number of neighbors, allowing individuals to make very informed decisions. Additionally, individuals with a high number of edges are able to see a lot of potential color changes. If the population is large and over-randomness is a concern, this is lessened when individuals can see a wide range of neighbors and will not randomly change color if they see one of their many neighbors changing. Thus, the system will be allowed to settle into the global solution, even when individuals playing random update rules are likely to choose a random color.

## Discussion

The 2-coloring problem, while trivial on a global scale, represents new challenges when solved by a population of agents that have only limited local information. When an agent only sees a small fraction of the entire network, they can be led astray into making myopic decisions that are non-optimal for the population at large. On the other hand, agents making random decisions, however infrequently, can serve to perturb a system that is stuck at a local minimum, thereby breaking up gridlock and moving the population toward the desired global coordination.

Among others, an important insight stemming from the present paper is that the type of decision update rule used by agents is at least as important as the amount of random behavior. The randomness-first and memory-*N* update rules require different conditions to be successful. This gives us two different update rules that are useful in different settings and should be thought of as complementary instead of one being superior to the other. For example, in a scenario where all agents are able to use a randomness-based update rule, a memory-*N* update rule can be used to great success. However, if only a few agents in the population can be persuaded to take on the personal risk of behaving randomly (or a small number of bots prescribed with random behavior have been introduced into the population like in the study by Shirado and Christakis 2017), a randomness-first update rule with a low *p* will have a higher chance of success.

### Limitations of the study

This paper most closely relates to previous work involving human subjects playing the coloring game with random bots (Shirado and Christakis 2017). While random behavior was observed coming from human players (Kearns 2006), it is not clear if this behavior was closer to the randomness-first or the memory-*N* update rule. The noisy bots themselves in the study by Shirado and Christakis 2017 played a randomness-first update rule, which may explain how such a small fraction (*ρ*_*r*_ = 0.15) of random actors had such a profound impact on the network coloring game. While the artificial agents in this work may not fully capture sophisticated human behavior, they indeed encompass the essence of random exploration ubiquitous in human decision-making, as demonstrated in prior observations of human decision choices in game theoretical interactions (Traulsen et al., 2009). It is thus promising for future work to leverage existing data such as in the study by (Shirado and Christakis 2017) to further validate and refine the stochastic decision update rules presented in this paper.

Our work demonstrates that the solving of challenging distributed network coloring problems can be achieved by entirely using myopic artificial agents without human subjects. We find that it is necessary to have enough randomness to ensure that the system is able to find the global coloring, but without having so much random behavior, the system never settles down. That said, certain randomness-based update rules can be particularly successful, depending on the underlying population characteristics (see Table 1). In this regard, our findings as summarized in Table 1 can be used to inform future hybrid experiment design.

**Table 1:** Population characteristics determine the efficacy of each decision update rule in solving collective network coloring problems.

Population characteristics	Optimal update rule
Small *ρ*_*r*_	Randomness-first update rule
Large *ρ*_*r*_, small *n*	Memory-0 update rule
Large *ρ*_*r*_, large *n*	Memory-*N* update rule, *N* ≥ 1

This table summarizes how ρ_r,_ the proportion of agents with random behavior, and *n*, the size of the population, can impact which stochastic update rule used by noisy agents will work best together with the remaining greedy agents.

Of particular note, here we only consider the simplest possible 2-colorings of bipartite networks, which is surely an over-simplification of the more general coloring problems. Introducing even one more color adds all sorts of complications. For example, the bowtie analysis completely falls apart, as the subgraphs to result in gridlock in a 3-colorable network are significantly larger and more complex. Besides, this paper also only considers populations that play a mix of two decision update rules: a fraction of the agents use greedy decision rule and the rest use randomness-based rule. It is possible that other potential combinations, such as a mixed population of agents using the randomness-first rule and the memory-*N* rule, could succeed in places where neither update rule succeeds alone. Future work taking into account these extensions will be of interest and improves our understanding of collective decision-making in the presence of noises (Couzin et al, 2005, 2011) and, more generally, machine behavior (Rahwan et al., 2019).

### Resource availability

#### Lead contact

Dr. Feng Fu, Address: 27 N. Main Street, 6188 Kemeny Hall, Department of Mathematics, Dartmouth College, Hanover, NH 03755, USA. Tel: +1 (603) 646 2293, Fax: +1 (603) 646 1312. Email: fufeng@gmail.com.

#### Materials availability

All materials related to this paper have been included in the paper.

#### Data and code availability

All simulation data have been included in the paper. The simulation code that can be used to reproduce the work is available at GitHub: https://github.com/MattJonesMath/distributed-network-coloring.
